# Impact of Remote Blood Pressure Monitoring Device Connectivity on Engagement Among Pregnant Individuals Enrolled in the Delfina Care Platform: Observational Study

**DOI:** 10.2196/55617

**Published:** 2024-07-12

**Authors:** Mia Charifson, Timothy Wen, Bonnie Zell, Priyanka Vaidya, Cynthia I Rios, C Funsho Fagbohun, Isabel Fulcher

**Affiliations:** 1 Delfina Care Inc, San Francisco, CA, United States; 2 Department of Population Health, New York University, New York, NY, United States; 3 Department of Obstetrics, Gynecology, and Reproductive Sciences, University of California San Francisco, San Francisco, CA, United States; 4 Center for Women's Health, Richmond, TX, United States

**Keywords:** blood pressure, hypertension, remote patient monitoring, pregnancy, digital health, remote monitoring, user engagement, users, connected, unconnected, comparison, patient engagement, prospective pregnancy cohort, device, devices, female, females, women, logistic regression, Poisson

## Abstract

User engagement with remote blood pressure monitoring during pregnancy is critical to optimize the associated benefits of blood pressure control and early detection of hypertensive disorders of pregnancy. In our study population of pregnant individuals, we found that *connected* blood pressure cuffs, which automatically sync measures to a monitoring platform or health record, increase engagement (2.13 [95% CI 1.36‐3.35] times more measures per day) with remote blood pressure monitoring compared to *unconnected* cuffs that require manual entry of measures.

## Introduction

Hypertensive disorders of pregnancy affect 5%‐10% of pregnancies and increase the risk of adverse pregnancy and postpartum outcomes [1,2]. Typically, management of these disorders involves blood pressure (BP) monitoring and initiation of antihypertensive therapy. Remote BP monitoring (RBPM) enables at-home BP monitoring to inform clinical decision-making in a timely manner [2-4]. Previous studies have shown that RBPM facilitates earlier detection of elevated BP and reduces prenatal hospitalizations and clinic visits [2,3]. Despite studies establishing feasibility and patient acceptability of RBPM [4-9], best practices for integrating RBPM within prenatal care have not yet been established. One open question is whether connected BP cuffs, which automatically sync measures to a health record, improve data quality and frequency over standard BP cuffs, which require users to manually enter measures [3-8]. The goal of this study was to compare user engagement with RBPM between *connected* and *unconnected* BP devices among users of a pregnancy care platform.

## Methods

### Study Population

The study population consisted of pregnant individuals enrolled in Delfina Care [10], a comprehensive pregnancy care platform, at a community practice in Texas, USA, between January and July 2023. Initially, these users were provided with unconnected BP devices at their provider’s discretion, with a recommendation to record 2 BP measures a day per internal expert clinical consensus. Connected devices were introduced in April 2023 as part of a quality improvement initiative. Differences in user experiences between the connected and unconnected device groups are further described in Multimedia Appendix 1.

The outcome of interest was user engagement, defined as the number of daily BP measures taken, and as a binary indicator of completing the daily recommended BP measures (ie, 2 distinct BP measures per day). Analogous engagement outcomes at the weekly level were also reported. To avoid inflated differences due to repeated measures from connected device users, we considered multiple entries within 1 hour as a single measurement (Multimedia Appendix 2). The exposure of interest was receiving a connected versus unconnected device. Clinical and demographic characteristics were collated from user-reported questionnaire data and electronic health records.

### Statistical Analysis

Poisson and logistic regressions were fit for the daily number of BP measures and ≥2 daily BP measures, respectively. Both models controlled for relevant confounders (ie, age, parity, weeks since enrollment, primary clinic, and preferred language) and included a random effect for users across enrollment days.

### Ethical Considerations

The study team received an institutional review board exemption waiver of HIPAA (Health Insurance Portability and Accountability Act) authorization on August 22, 2022, from WIRB-Copernicus Group (WCG) Institutional Review Board (protocol number 202208‐001).

## Results

During the study period, 164 users with BP cuffs were enrolled in the Delfina Care platform. Restricting to those with covariate data, the analytic sample consisted of 163 users (97 unconnected device users and 66 connected device users). Users with connected devices had more mean BP measure entries per day (0.51 vs 0.32) and a higher proportion completed the recommended ≥2 daily BP measures (12% vs 7%) compared to users with unconnected devices (Table 1). At the weekly level, the mean entries per week (3.37 vs 2.07) and the proportion of users who completed the recommended ≥1 weekly BP measure (63% vs 47%) were higher for connected users than for unconnected device users (Table 1).

Adjusting for confounders, users with connected devices had 2.13 (95% CI 1.36‐3.35) times more measures per day and 5.62 (95% CI 2.28‐13.83) times the odds of meeting the recommendation of ≥2 daily BP measures than unconnected device users (Table 2).

**Table 1.** Study population characteristics.

Characteristics	Overall (n=163)	Unconnected device users (n=97)	Connected device users (n=66)	*P* value
Age in years, mean (SD)	28.73 (6.02)	28.71 (5.61)	28.76 (6.61)	.96
**Language, n (%)**				.32
English	139 (85.3)	80 (82.5)	59 (89.4)	
Spanish	24 (14.7)	17 (17.5)	7 (10.6)	
Parity, mean (SD)	1.37 (1.44)	1.36 (1.32)	1.38 (1.60)	.99
**Primary clinic, n (%)**				.07
A	35 (21.5)	20 (20.6)	15 (22.7)	
B	71 (43.6)	49 (50.5)	22 (33.3)	
C	57 (35.0)	28 (28.9)	29 (43.9)	
**User engagement^a^, mean (SD)**				
Weekly entries	2.59 (3.02)	2.07 (2.78)	3.37 (3.20)	.007
Daily entries	0.40 (0.46)	0.32 (0.42)	0.51 (0.49)	.008
Proportion ≥1 weekly entries	0.53 (0.35)	0.47 (0.35)	0.63 (0.33)	.003
Proportion ≥2 daily entries	0.09 (0.17)	0.07 (0.15)	0.12 (0.19)	.048

a User engagement metrics were first averaged within a user and then averaged across users for each device type and overall.

**Table 2.** Effect estimates and 95% CIs by outcome model.

	Number of BP measures per day		Completed ≥2 BP measures per day	
Predictors	Incidence rate ratio	95% CI	Odds ratio	95% CI
Connected versus unconnected device	2.13	1.36‐3.35	5.62	2.28‐13.83
Age (years)	1.06	1.02‐1.11	1.11	1.03‐1.20
Weeks since enrollment	0.97	0.97‐0.98	1.00	0.98‐1.01
English vs Spanish language	1.25	0.66‐2.35	0.72	0.22‐2.38
Parity (number of live births)	0.88	0.74‐1.05	0.79	0.57‐1.10
Clinic B vs A	1.44	0.79‐2.61	4.18	1.25‐14.03
Clinic C vs A	1.54	0.83‐2.86	4.06	1.16‐14.20

## Discussion

We observed user engagement with RBPM was significantly higher among those with connected devices than those with unconnected devices. Previous studies among pregnant individuals have shown that the recommended frequency of BP measures ranges from several times daily to once weekly [4,7,8,11]. In our cohort, the proportion of users meeting the twice-daily recommendation was low, but the majority of users completed the once-weekly entry at least, and connected device users still had higher utilization than unconnected device users (63% vs 47%; *P*=.003). Compared to in-clinic BP measures, all users had a higher average number of weekly readings than the in-clinic average (2.59 vs 0.50 BP readings/week). Our study corroborated the feasibility of at-home RBPM during pregnancy and highlights the potential advantages of device connectivity on user engagement.

### Limitations

Our findings are limited by the lack of true randomization to device types, which we addressed by controlling for potential confounders. We also addressed potential time-related confounding via a sensitivity analysis with no change in findings (Multimedia Appendix 2).

### Conclusion

This study highlights how connected BP devices can improve patient engagement to RBPM during the prenatal period. Other aspects of RBPM, such as recommended frequency and patient education, should be further investigated to ensure users are able to successfully engage with monitoring technologies.

## Supplementary material

10.2196/55617 Multimedia Appendix 1 Additional information on Delfina Care.

10.2196/55617 Multimedia Appendix 2 Additional analyses.

## Abbreviations

BP: blood pressure
HIPAA: Health Insurance Portability and Accountability Act
NICHD: National Institute of Child Health and Human Development
RBPM: remote blood pressure monitoring
WCG: WIRB-Copernicus Group
