# Supplementary material for: Impact of Remote Blood Pressure Monitoring Device Connectivity on Engagement Among Pregnant Individuals Enrolled in the Delfina Care Platform: Observational Study
Source: JMIR Mhealth Uhealth. 2024 Jul 12;12:e55617. doi: 10.2196/55617 (PMC11259580; doi:10.2196/55617)
Supplement: Multimedia Appendix 1 [file mhealth-v12-e55617-s001.docx]

**Multimedia Appendix 1: Additional information on Delfina Care**

**
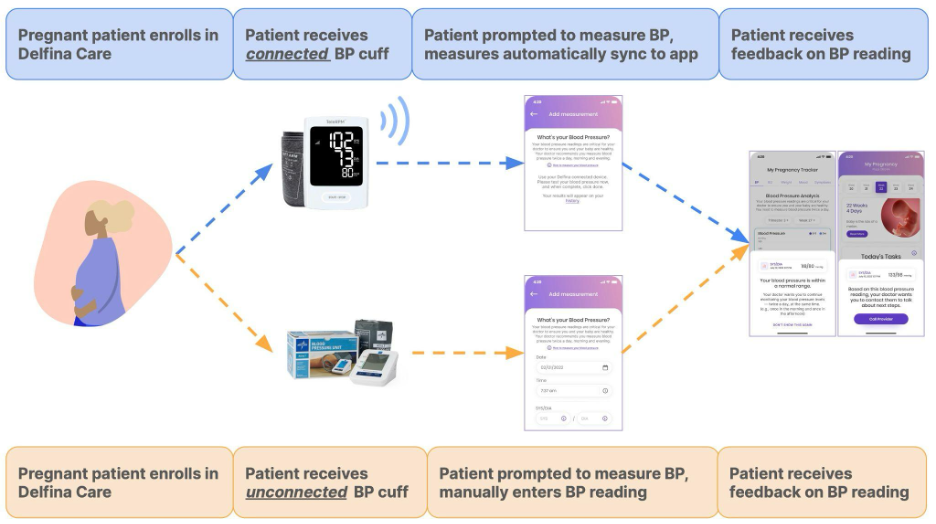
**

Delfina Care platform is an integrated pregnancy platform for patients to track their health and stay connected with their providers during pregnancy. Delfina Care launched at the Texas community practice in January 2023 at no cost to patients as a pilot program for the platform. At a prenatal visit, patients were invited to enroll in Delfina Care. If interested in enrollment, patients downloaded the Delfina Care smartphone application (app) and providers screened patients to determine if they may benefit from an at-home blood pressure cuff or a glucometer. Providers monitored blood pressure entries from a provider dashboard developed by Delfina.

Blood pressure cuffs were all Smart Meter devices. At first, patients were only provided the unconnected cuff, but starting in April 2023, the connected cuffs were introduced. The connected cuffs are WiFi-enabled, allowing them to transmit measurements directly into the app without manual entry. The above figure visualizes the patient experience between connected (blue pathway) and unconnected (orange pathway) device users. At enrollment, patients were instructed by medical staff at their enrollment visit on how to use the cuffs to take their blood pressure at home. At home, both patient groups utilize the app to view blood pressure trends and can set reminders for BP measurement. However, unconnected BP cuffs users need to manually enter their BP readings into the app, whereas connected BP cuffs automatically sync measurement to app and provider dashboard. After recording a BP measure via either device, patients then receive a feedback screen regarding their reading.

Along with blood pressure, patients can record other health features in the app throughout pregnancy (weight, blood sugar, symptoms, and mood) as well as receive educational material tailored to their gestational age, communicate/schedule meetings with their providers, and schedule one-on-one meetings with Delfina guides, all trained doulas. Patients receive in-app feedback regarding their health tracking in line with ACOG guidelines.
