# Supplementary material for: Impact of Remote Blood Pressure Monitoring Device Connectivity on Engagement Among Pregnant Individuals Enrolled in the Delfina Care Platform: Observational Study
Source: JMIR Mhealth Uhealth. 2024 Jul 12;12:e55617. doi: 10.2196/55617 (PMC11259580; doi:10.2196/55617)
Supplement: Multimedia Appendix 2 [file mhealth-v12-e55617-s002.docx]

**Multimedia Appendix 2: Additional analyses**

Repeat measures: Compared to 52% (50/97) of unconnected device users, 80% (53/66) of connected device users had at least one repeat measure. To account for duplicate entries in the analysis, we considered multiple entries within one hour as one measurement. However, the high number of repeat measures observed among connected device users may suggest that connected devices increase the number of measures taken or entered in one sitting. Alternatively, patients may have been unaware of device connectivity and manually logged connected measures causing entry duplication. For the future, we suggest improving patient education around device connectivity to avoid duplicate entries.

Sensitivity analysis: Efforts to increase patient engagement with Delfina Care coincided with when connected devices were introduced. To address the potential for spurious association between device type and patient engagement, we conducted a sensitivity analysis restricting to only observations collected after connected devices were introduced (April 25, 2023). A sensitivity analysis restricted to all observations after connected devices were introduced were consistent with our main results, although attenuated with respect to meeting the recommended ≥2 daily BP measures (2.89 [95% CI: 0.99-8.43]).
